# Report of Mortality in Buffalo for the Month February, 1856

**Published:** 1856-04

**Authors:** C. L. Dayton

**Affiliations:** Health Physician


ART. V.—*Report of Mortality in Buffalo for the month February*, 1856. By C. L. Dayton, M. D., Health Physician.

| DISEASES. | No. | Males. | Females. |
|---|---|---|---|
| Accidents, | 2 | .... | 2 |
| Cynanche Trachealis, | 3 | 1 | 2 |
| Carcinoma Maxilare Inferius, | 1 | 1 | .... |
| Cystitis, | 1 | 1 | .... |
| Eclampsia, | 13 | 8 | 5 |
| Enteritis, | 4 | 3 | 1 |
| Erysipelas, | 1 | .... | 1 |
| Exposure and Abandonment, | 1 | .... | 1 |
| Febris Typhoid, | 2 | .... | 2 |
| " Scarlatina, | 1 | .... | 1 |
| " Gastrica, | 1 | 1 | .... |
| " Rheumatica, | 1 | ... | 1 |
| " Congestiva, | 1 | 1 | .... |
| Gangrena Pulmonalis, | 1 | .... | 1 |
| Gastritis, | 1 | 1 | .... |
| Hydrocephalus, | 4 | 2 | 2 |
| Hydrothorax, | 1 | 1 | .... |
| Hæmorrhagia Uterina, | 2 | .... | 2 |
| Hæmorrhagia Intestinorum, | 2 | 1 | 1 |
| Intersusception, | 1 | .... | 1 |
| Murder, | 1 | 1 | .... |
| Marasmus, | 1 | .... | 1 |
| Meningitis, | 2 | 1 | 1 |
| Morbus Cordis, | 2 | 1 | 1 |
| Negligentia, | 1 | 1 | .... |
| Peritonitis, | 1 | 1 | .... |
| Pneumonitis, | 8 | 4 | 4 |
| " Typhoides, | 1 | 1 | .... |
| Pertussis, | 2 | 2 | .... |
| Paralysis Pulmonalis, | 1 | 1 | .... |
| Rubeola, | 1 | ... | 1 |
| Rheumatic Metastasis, | 1 | 1 | .... |
| Senectus, | 2 | 2 | ... |
| Tabes Mesenterica, | 1 | 1 | .... |
| Teething, | 3 | 1 | 2 |
| Tuberculosis Pulmonalis, | 15 | 8 | 7 |
| Ignotus, | 24 | 14 | 5 |
| Totals, | 111 | | |

SEXES.

| | |
|---|---|
| Males, | 61 |
| Females, | 45 |
| Sex not given, | 5 |
| Total, | 111 |

REGISTER OF MORTALITY—Continued.

AGES.

| | | | |
|---|---|---|---|
| Still-born, | 7 | Between 10 years and 20 years, | 6 |
| 1 day, | 2 | " 20 " " 30 " | 11 |
| Between 1 day and 30 days, | 10 | " 30 " " 40 " | 9 |
| " 1 month and 6 months, | 16 | " 40 " " 50 " | 6 |
| " 6 months and 12 " | 11 | " 50 " " 60 " | 5 |
| " 1 year " 3 years, | 19 | " 60 " " 70 " | 1 |
| " 3 " " 5 " | 2 | " 70 " " 80 " | 1 |
| " 5 " " 10 " | 4 | " 90 " " 100 " | 1 |
| Total number under 10 years, | 71 | Total number over 10 years, | 40 |

NATIVITIES.

| | | | |
|---|---|---|---|
| American, (white, 55) colored, 2, | 57 | French, | 2 |
| German, | 26 | Swiss, | 1 |
| Irish, | 8 | Norwegian, | 1 |
| English, | 3 | Country not given, | 12 |
| Canadian, | 1 | | |
| Total, | | | 111 |

